# Characteristics, Location, and Clinical Outcomes of Gastrointestinal Bleeding in Patients Taking New Oral Anticoagulants Compared to Vitamin K Antagonists

**DOI:** 10.3390/jcm10122693

**Published:** 2021-06-18

**Authors:** A Reum Choe, Chang Mo Moon, Chung Hyun Tae, Jaeyoung Chun, Ki Bae Bang, Yoo Jin Lee, Hyun Seok Lee, Yunho Jung, Sung Chul Park, Hoon Sup Koo

**Affiliations:** 1 Department of Internal Medicine, College of Medicine, Ewha Womans University, Seoul 07985, Korea; prems0121@naver.com (A.R.C.); jhtae@ewha.ac.kr (C.H.T.); 2 Inflammation-Cancer Microenvironment Research Center, College of Medicine, Ewha Womans University, Seoul 07804, Korea; 3 Department of Internal Medicine, Seoul National University College of Medicine, Liver Research Institute, Seoul 03080, Korea; J40479@gmail.com; 4 Department of Internal Medicine, Gangnam Severance Hospital, Yonsei University College of Medicine, Seoul 06273, Korea; 5 Department of Internal Medicine, Dankook University College of Medicine, Cheonan 31116, Korea; kibaebang@gmail.com; 6 Department of Internal Medicine, Keimyung University School of Medicine, Daegu 42601, Korea; doctorlyj@dsmc.or.kr; 7 Department of Internal Medicine, School of Medicine, Kyungpook National University, Kyungpook National University Hospital, Daegu 41404, Korea; lhsworld@nate.com; 8 Department of Internal Medicine, Soonchunhyang University College of Medicine, Cheonan 31151, Korea; yoonho7575@naver.com; 9 Department of Internal Medicine, Kangwon National University School of Medicine, Chuncheon 24289, Korea; schlp@hanmail.net; 10 Department of Internal Medicine, Konyang University College of Medicine, Daejeon 35365, Korea; koo7574@naver.com

**Keywords:** gastrointestinal bleeding, new oral anticoagulants, vitamin K antagonist, rebleeding

## Abstract

New oral anticoagulants (NOACs) are commonly used in clinical practice as alternatives to vitamin K antagonists (VKA). However, the etiology, clinical course, and risk of gastrointestinal (GI) bleeding remain unclear. We aimed to evaluate the clinical characteristics and location of acute GI bleeding associated with NOACs and its severity and outcomes compared to VKA. This retrospective multicenter study included 381 subjects on anticoagulants who underwent appropriate diagnostic examination due to GI bleeding. Regarding the characteristics of acute GI bleeding, the proportion of vascular lesions was significantly lower in the NOACs group than that in the VKA group. Small bowel bleeding occurred less commonly in the NOACs group, but the difference did not reach statistical significance. Regarding severity and clinical outcomes, patients on NOACs received significantly smaller volumes of transfused blood products and had shorter ICU stays than those on VKA. Moreover, the need for surgery and the risk of rebleeding in the NOACs group were significantly lower than those in the VKA group. Patients on NOACs have better clinical outcomes in terms of severity of acute GI bleeding or rebleeding than patients on VKA. Patients on NOACs demonstrate different characteristics and location of acute GI bleeding than those on VKA.

## 1. Introduction

Since the Food and Drug Administration (FDA) approved new oral anticoagulants (NOACs) in 2010 [1,2], direct factor Xa inhibitors (rivaroxaban, apixaban, and edoxaban) and direct thrombin inhibitors (dabigatran) are now available in clinical practice [3,4]. The 2016 European Society of Cardiology guidelines recommended NOACs for patients with non-valvular atrial fibrillation (NVAF) to prevent stroke [5]. The 2016 American College of Chest Physician guideline and expert panel report also suggested a prescription in favor of NOACs to vitamin K antagonist (VKA) for the initial and long-term management of venous thromboembolism in patients without cancer [6].

The VKA inhibits vitamin K epoxide reductase, thereby attenuating the reduction of oxidized vitamin K in the liver. In contrast to VKA, the NOACs directly inhibit a single clotting enzyme; dabigatran inhibits thrombin, whereas rivaroxaban, apixaban, and edoxaban inhibit factor Xa [7,8]. The NOACs have major pharmacologic advantages over VKA, including fast onset/offset of action, few clinically relevant interactions with other drug and food, and predictable pharmacokinetics, simple administration by fixed doses without any monitoring [9,10,11].

Recently, several randomized clinical trials have shown that NOACs is preferred to VKA, due to its efficacy in preventing stroke and systemic embolisms in patients with NVAF [12,13,14]. NOACs have been reported to significantly decrease the prevalence of major bleeding, particularly the rates of intracranial hemorrhage and critical bleeding [4,15]. Moreover, several meta-analyses have shown that NOACs have a more favorable safety profile than VKA [16,17,18,19]. However, the risk of NOAC-associated bleeding, particularly gastrointestinal (GI) bleeding, is still a concern. The ROCKET AF trial [20], a comparative study of rivaroxaban and warfarin for the prevention of stroke and embolism, showed that patients treated with rivaroxaban had a significantly higher rate of GI bleeding than those treated with VKA. Contrarily, the XANTUS registry [21] investigated the stroke prevention effect of anticoagulants in patients with AF and showed that major GI bleeding occurred less frequently in the rivaroxaban group. To date, it remains unclear whether NOACs increases the risk of GI bleeding compared to warfarin. Moreover, few studies have reported the exact source and location of GI bleeding during NOACs treatment with comprehensive examination methods, including gastrointestinal endoscopy or abdominal pelvis computed tomography (CT).

Therefore, we aimed to assess the clinical and endoscopic features of acute GI bleeding in patients prescribed NOACs and evaluate the severity and clinical outcomes of these events compared to VKA.

## 2. Materials and Methods

### 2.1. Study Population

**Figure 1:**
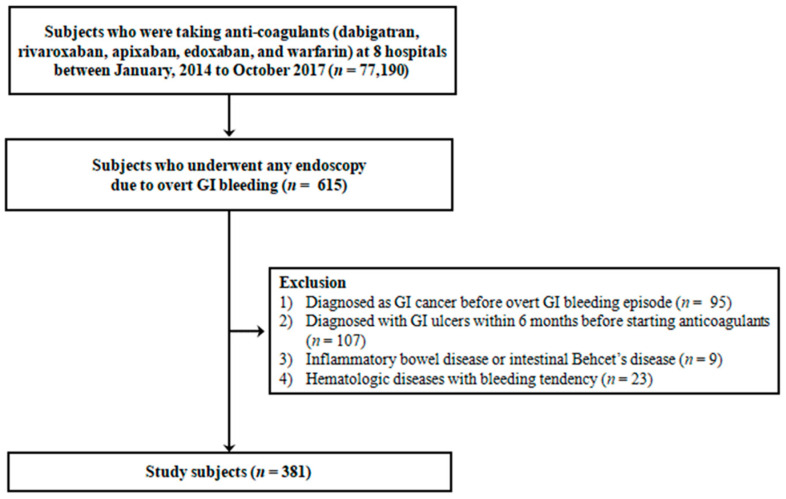
Flow diagram. A total of 615 patients who underwent any endoscopy due to overt GI bleeding were enrolled from eight large-volume university hospitals. Of these, 234 patients were excluded, and 381 patients were enrolled for analysis.

In this retrospective multicenter cohort study, we analyzed the clinical data of study subjects collected at eight tertiary medical institutions between January 2014 and October 2017 in the Republic of Korea. We included subjects who met the following three criteria: (1) patients who visited the hospital with symptoms of overt GI bleeding; (2) patients treated with anticoagulants (dabigatran, rivaroxaban, apixaban, edoxaban, and warfarin) for at least 3 months; (3) patients who underwent diagnostic esophagogastroduodenoscopy (EGD), colonoscopy, sigmoidoscopy, small bowel (SB) enteroscopy, or capsule endoscopy to identify the focus of GI bleeding, according to the diagnostic strategy of each hospital. Subjects were excluded in the following conditions: (1) those diagnosed with GI cancer before overt GI bleeding episode (*n* = 95); (2) GI ulcers within 6 months before starting anticoagulants (*n* = 107); (3) inflammatory bowel disease or intestinal Behçet’s disease (*n* = 9); and (4) hematologic diseases with a bleeding tendency (*n* = 23). Finally, a total of 381 patients were included in this study (Figure 1). The study protocol conforms to the ethical guidelines of the 1975 Declaration of Helsinki as reflected in a priori approval by the institution’s human research committee of all participating hospitals. 

### 2.2. Data Collection and Definition of Variables

We collected the demographic, clinical, and laboratory data from the patients at the time of presentation. The baseline characteristics included the presence of major GI bleeding, history of prior GI bleeding, indication for anticoagulation, medical comorbidities, and any concomitant drugs associated with GI bleeding. The risk of major bleeding was calculated using the HAS-BLED (old age, drugs/alcohol intake, hypertension, abnormal liver/kidney function, stroke, bleeding predisposition or history, and labile international normalized ratio) scoring system including six comorbid conditions.

GI bleeding was identified from the medical records by the presence of hematemesis, melena, or hematochezia. Major bleeding was defined as fatal or symptomatic bleeding in a critical organ or bleeding that caused a decrease in hemoglobin level of 2 g/dL or more, leading to transfusion of 2 or more units of whole or red blood cells [22]. Location of GI bleeding was identified as upper GI, small bowel, lower GI, or indeterminate by reviewing endoscopic or radiologic records. The diagnostic modalities for identifying the causes of GI bleeding included EGD, colonoscopy/sigmoidoscopy, SB enteroscopy, capsule endoscopy, or abdominal pelvic computerized tomography (CT).

GI bleeding lesions were divided into four types according to the endoscopic characteristics: (1) vascular lesion (angiodysplasia, Dieulafoy’s lesion, varices, gastric antral vascular ectasia, hemorrhoid, and ischemic colitis); (2) inflammatory lesion (esophagitis, gastritis, colitis, erosion, ulcer, and inflammatory bowel disease); (3) neoplastic lesion (polyp, tumor); (4) anatomic lesion and others (diverticulum, Mallory–Weiss syndrome, post-procedural bleeding after polypectomy, or endoscopic submucosal dissection).

Clinical outcomes were investigated by hemodynamic instability at the point of admission, need for angiographic or surgical intervention, in-hospital mortality, and rebleeding. Hemodynamic instability was defined as one or more out-of-range vital sign measurements, such as systolic blood pressure < 90 mmHg or heart rate > 100/min. Rebleeding was defined as endoscopic confirmation of newly developed GI bleeding or an explained drop in hemoglobin more than 2 g/dL after 7 days of initial endoscopic hemostasis treatment [23,24].

### 2.3. Statistical Analysis

Continuous variables were presented as mean ± standard deviation, and categorical variables were presented as the number of subjects and percent. Group comparison was performed by using independent-samples t-tests or Mann–Whitney U-tests for continuous variables and Pearson’s chi-squared tests or Fisher’s exact tests for categorical variables. The adjusted odds ratio for clinical outcomes was obtained by multivariable logistic-regression analysis adjusted for sex and HAS-BLED score. Any variable with a *p*-value < 0.2 in univariate analysis was accepted as a candidate for multivariate analysis along with variables with known clinical importance. Finally, statistical significance was considered as *p* < 0.05 with a two-tailed test. We used the analysis of covariance for the number of red blood cell transfusions, days in the hospital, and ICU days. The analyses were adjusted for sex and HAS-BLED score as continuous variables. All statistical analyses were performed using SPSS for Windows version 21.0 (SPSS Inc., Chicago, IL, USA).

## 3. Results

### 3.1. Baseline Characteristics of Study Subjects

The baseline characteristics of the patients on NOACs or VKA who experienced acute GI bleeding are shown in Table 1. Among them, 144 patients were prescribed NOACs, and 237 patients used VKA (mean age; 77.9 ± 7.8 vs. 73.3 ± 11.9 years). Regarding indications for anticoagulation, NOACs were used for AF or atrial flutter in 108 cases (75.0%) and pulmonary embolism or deep vein thrombosis in 29 cases (20.1%). VKAs were used for AF or atrial flutter in 117 cases (49.4%) and prosthetic valves in 69 cases (29.1%). Twenty-five of 144 (17.3%) patients on NOACs concomitantly had antiplatelet agents (aspirin, clopidogrel), whereas 36 of 237 (15.2%) on VKA used antiplatelet agents. The concomitant use of proton pump inhibitor did not differ significantly between the two groups, while the use of H2 receptor antagonist showed more common in NOACs group. There was no difference in examination modalities between the two groups.

### 3.2. Source, Lesion, and Location of Acute GI Bleeding in Patients on NOACs or VKA

The most common site of acute GI bleeding was the upper GI tract in the NOACs (51/144, 35.4%) and the VKA group (98/237, 41.4%). Small bowel bleeding was observed in 6/144 (4.2%) in the NOACs group and 16/237 (6.8%) in the VKA group. The prevalence of lower GI bleeding was 33/144 (22.9%) in the NOACs group and 43/237 (18.1%) in the VKA group.

Among the 90 patients on NOACs who experienced GI bleeding, the common causes of upper GI bleeding were benign gastric ulcer in 25 (27.8%) patients, duodenal ulcer in 5 (5.6%), gastric varix in 3 (3.3%), and Mallory–Weiss syndrome in 3 (3.3%) patients. The common causes of small bowel bleeding were vascular lesions in 4 (4.4%) and inflammatory lesions in 2 (2.2%) patients. The common causes of lower GI bleeding were rectal ulcer without exposed vessels in 8 (8.9%) patients, diverticuli without current bleeding in 7 (7.8%), and colon polyp bleeding in 5 (5.6%) patients. Among the 157 patients on VKA who experienced GI bleeding, the common causes of upper GI bleeding were benign gastric ulcer in 47 (29.9%) patients, duodenal ulcer in 14 (8.9%), and gastric angiodysplasia in 9 (5.7%) patients. The common causes of small bowel bleeding were inflammatory lesions in 9 (5.7%) and vascular lesions in 6 (3.8%) patients. The common causes of lower GI bleeding were hemorrhoid bleeding in 10 (6.4%) patients, colon polyp bleeding in 10 (6.4%), rectal ulcer without exposed vessels in 4 (2.5%), and diverticuli without current bleeding in 4 (2.5%) patients (Table 2).

Regarding the characteristics of GI bleeding in the two groups, the proportion of vascular lesions in the location of GI bleeding, bleeding in the small bowel occurred less commonly in patients on NOACs, but the difference could not reach statistical significance (6.7% vs. 10.2%, *p* = patients on NOACs was significantly lower than in those patients on VKA (15.6% vs. 25.5%, *p* = 0.038). Regarding 0.090) (Table 3).

### 3.3. Comparison of Clinical Outcomes in Patients on NOACs vs. VKA

**Figure 2:**
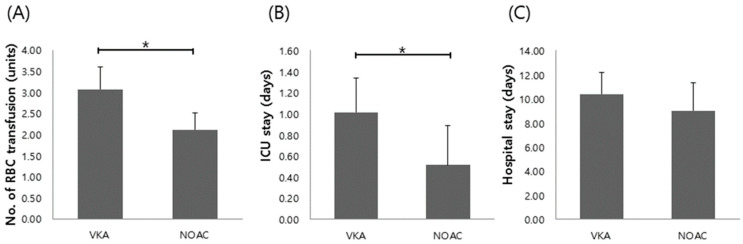
Clinical outcomes related to the severity of GI bleeding in patients on NOACs vs. VKA (**A**) number of red blood cell transfusion, (**B**) duration of ICU stay, (**C**) duration of hospital stay in patients treated with VKA and NOACs. * *p* < 0.05. VKA, vitamin K antagonist; NOAC, new oral anticoagulants; RBC, red blood cell; ICU, intensive care unit.

Regarding clinical outcomes, patients treated with NOACs received significantly smaller volumes of blood transfusions with packed red blood cells than those taking VKA (2.1 ± 0.3 vs. 3.1 ± 0.2, *p* = 0.009). Patient treated with NOACs stayed in ICU significantly shorter than those taking VKA (0.5 ± 0.2 vs. 1.0 ± 0.2, *p* = 0.049). However, there was no significant difference in the stay of hospital between patients treated NOACs and VKA (9.0 ± 1.2 vs. 10.4 ± 0.9, *p* = 0.344) (Figure 2).

In multivariate analysis adjusted for sex and HAS-BLED scores, rebleeding was less common in patients on NOACs than in those on VKA (adjusted OR 0.42, 95% CI 0.22–0.79, *p* = 0.007). Regarding the need for surgery, a very low number of patients required a surgical intervention in both group (1 case in NOAC group and 4 cases in VKA group). There was no significant difference in hemodynamic instability at admission, the need for angiography, and mortality during hospitalization between the two groups (Table 4).

We analyzed the clinical outcomes in the patients associated with different NOACs such as dabigatran, rivaroxaban, apixaban, and edoxaban. Consequently, unfavorable clinical outcomes such as hemodynamic instability at admission, need for angiography or surgery, mortality during hospital days, and rebleeding were the most frequent in those with rivaroxaban compared with other NOACs (Table 5).

## 4. Discussion

In the present study, patients treated with NOACs who experienced acute GI bleeding had different characteristics and clinical outcomes than those treated with VKA. The proportion of vascular lesions and small bowel bleeding was lower in the NOACs group than that in the VKA group. The clinical outcomes in terms of severity and rebleeding are better in the NOACs group than in the VKA group.

Patients on NOACs who experienced GI bleeding had fewer unfavorable outcomes such as critical bleeding events requiring blood transfusion or rebleeding than those on VKA. Our results suggest that acute GI bleeding associated with NOACs may be less severe than that associated with VKA, which may be explained by the short half-life of NOACs (NOACs around 8–14 h, VKA 36–42 h) [1,25]. Therefore, the cessation of NOACs leads to a return of the coagulant function and recovery in a short period [26]. If GI bleeding is recognized, discontinuation of NOACs can quickly attenuate their anticoagulation effect. Moreover, this difference in the results achieved with NOACs and VKA was due to the potentially dangerous overdosing of VKA, which frequently occurs in clinical settings [27,28,29]. VKA have a large number of food or drug interactions, which complicate its anticoagulation effect [30]. Especially, acute illness such as infection and organ failure can prolong the international normalized ratios (INRs) in patients on VKA [31]. The intrinsic difficulty in maintaining therapeutic levels in those treated with VKA results in supra-therapeutic INRs and a risk of severe bleeding [32]. Therefore, the difference in severity and outcomes of acute GI bleeding between NOACs and VKA may be explained by their pharmacological properties.

In this study, regarding the location of GI bleeding, bleeding in the small bowel occurred less common in patients on NOACs, but the difference could not reach statistical significance. Generally, bleeding in the small bowel remains relatively rare, accounting for 5–10% of all patients with GI bleeding [33]. Bleeding originated from the small bowel in 6 (6.7%) patients on NOACs and 16 (10.2%) patients on VKA in our study. Likewise, Diamantopoulou, et al. presented that the site of bleeding was located in the small bowel in 2/43 of NOAC patients and 6/68 of warfarin group [34]. Another cohort study also reported that GI bleeding associated with the use of dabigatran was more common from a source distal to the ligament of Treitz [35]. The pathophysiological explanation may relate to a low bioavailability of dabigatran [36]. Despite the similar mode of action, bioavailability differs according to the NOACs (dabigatran, 3–7%; apixaban, 50–60%; edoxaban, 62%; rivaroxaban 66–100%). The incidence of small bowel bleeding varies depending on the type or dosage of NOACs. This difference in results may be influenced by the type or dosage of NOACs and the characteristics of the study subjects. Therefore, further large-scale prospective studies are warranted to evaluate small bowel bleeding between these four NOACs.

In our cohort, vascular lesions were less common in patients on NOACs than in those on VKA. Pathophysiologically, NOACs is a non-absorbed, active anticoagulant within the GI tract lumen and promotes GI bleeding from vulnerable mucosal erosions [37]. Considering this characteristic, the use of NOACs may have no significant effect on intact mucosal lesions such as hemorrhoids, but can trigger bleeding in vulnerable mucosal lesions such as erosions or ulcers. These results may help to predict and prevent acute GI bleeding and evaluate the patients’ existing GI conditions before prescribing anticoagulants. In a recent network meta-analysis, apixaban had the highest probability to be the safest option with regard to the risk of GI bleeding, followed by edoxaban, warfarin, dabigatran, and rivaroxaban [38].

Our study has limitations. First, this study was conducted in an observational and retrospective manner, which may limit the generalization of its results and cause potential bias. It is impossible to completely control confounding factors such as comorbidities and medications that can affect acute GI bleeding. However, we tried to reduce this effect by adjusting for sex and HAS-BLED scores as confounding variables in our multivariate analysis. Second, diagnostic tests for GI bleeding such as EGD, colonoscopy, sigmoidoscopy, capsule endoscopy, SB enteroscopy, and abdomen pelvis CT were not equally performed in all patients. Also, some diagnostic modalities were not conducted in some subjects. However, as the eight institutions participating in this study were tertiary referral hospitals, the diagnostic strategy for acute overt GI bleeding was relatively similar. Third, due to the retrospective study design, there was a limitation in analyzing the acute changes just before GI bleeding, which could affect events.

Despite these limitations, our study had the following advantages. It showed the source of acute GI bleeding in NOACs, examined by endoscopic and imaging modalities. Moreover, we compared the clinical severity and outcomes of acute GI bleeding between NOACs and VKA by analyzing a relatively large amount of patient data.

## 5. Conclusions

Acute GI bleeding in patients on NOACs showed favorable clinical outcomes, such as the need for transfusion or surgery and rebleeding than in patients on VKA. Further, the characteristics and location of acute GI bleeding lesions differed between the NOACs and VKA group. Our results may help to determine the diagnostic and therapeutic approaches when physicians encounter acute GI bleeding events in patients on anticoagulants.

## Figures and Tables

**Table 1:** Baseline characteristics of the patients prescribed with NOACs or VKA who experienced GI bleeding.

	NOACs	VKA	*p* Value
	(*n* = 144)	(*n* = 237)	
Mean age, years (range) *	77.9 ± 7.8 (54–95)	73.3 ± 11.9 (29–95)	<0.001
Male sex (%)	63 (43.8%)	122 (51.5%)	0.071
Mean body mass index *	23.3 ± 3.8	22.1 ± 4.1	0.005
History of smoking (%)			0.187
No	124 (86.1%)	186 (78.5%)	
Ex-smoker	15 (10.4%)	38 (16.0%)	
Current smoker	5 (3.5%)	13 (5.5%)	
History of alcohol intake (%)			0.368
No	117 (81.3%)	198 (83.5%)	
Social	14 (9.7%)	26 (11.0%)	
Heavy	13 (9.0%)	13 (5.5%)	
History of major bleeding ^†^ (%)	17 (11.8%)	26 (11.0%)	0.903
History of prior gastrointestinal bleeding (%)	29 (20.1%)	42 (17.7%)	0.678
Symptom (%)			0.061
Hematemesis	25 (17.4%)	43 (18.1%)	
Melena	60 (41.7%)	124 (52.3%)	
Hematochezia	59 (41.0%)	70 (29.5%)	
Indication for Anticoagulation (%)			
Atrial fibrillation/flutter	108 (75.0%)	117 (49.4%)	<0.001
Pulmonary embolism/DVT	29 (20.1%)	40 (16.9%)	0.329
Prosthetic valve	1 (0.7%)	69 (29.1%)	<0.001
Stroke prevention	6 (4.2%)	11 (4.6%)	0.533
Comorbidities (%)			
Congestive heart failure	49 (34.0%)	77 (32.5%)	0.954
Hypertension	100 (69.4%)	137 (57.8%)	0.071
Arrythmia	108 (75.0%)	144 (60.8%)	0.019
Diabetes mellitus	53 (36.8%)	74 (31.2%)	0.362
Dyslipidemia	31 (21.5%)	42 (17.7%)	0.460
Coronary heart disease	29 (20.1%)	38 (16.0%)	0.394
Stroke	52 (36.1%)	58 (24.5%)	0.028
History of transient ischemic attack	4 (2.8%)	3 (1.3%)	0.314
Chronic kidney disease	14 (9.7%)	53 (22.4%)	0.001
Chronic obstructive pulmonary disease	6 (4.2%)	5 (2.1%)	0.273
Chronic hepatitis	1 (0.7%)	8 (3.4%)	0.086
Liver cirrhosis	13 (9.0%)	21 (8.9%)	0.955
Pulmonary embolism/DVT	26 (18.1%)	32 (13.5%)	0.297
Peripheral arterial occlusive disease	3 (2.1%)	13 (5.5%)	0.094
Prosthetic valve	2 (1.4%)	74 (31.2%)	<0.001
Concomitant medications (%)			
Aspirin	13 (9.0%)	27 (11.4%)	0.135
Clopidogrel	12 (8.3%)	9 (3.8%)	0.173
NSAIDs	5 (3.5%)	18 (7.6%)	0.080
Steroid	7 (4.9%)	15 (6.3%)	0.474
Proton pump inhibitor	29 (20.1%)	35 (14.8%)	0.233
H2 receptor antagonist	18 (12.5%)	10 (4.2%)	0.004
Examination Modalities (%)			
Esophagogastroduodenoscopy	43 (21.0%)	52 (16.0%)	0.116
Colonoscopy/Sigmoidfibroscopy	91 (44.4%)	160 (49.2%)	0.269
SB enteroscopy	0 (0.0%)	3 (0.9%)	0.294
Capsule endoscopy	12 (5.9%)	24 (7.4%)	0.591
Abdomen pelvis CT	59 (28.8%)	86 (26.5%)	0.440

NOACs, new oral anticoagulants; VKA, vitamin K antagonist; GI, gastrointestinal; DVT, deep vein thrombosis; NSAIDs, non-steroidal anti-inflammatory drugs; SB, small bowel; CT, computerized tomography; * Mean ± standard deviation; ^†^ History of major bleeding defined by International Society on Thrombosis and Hemostasis as fatal bleeding or symptomatic bleeding in a critical organ, or bleeding causing a decrease in hemoglobin level of 2 g/dL or more, leading to transfusion of 2 or more units of whole blood or red blood cells.

**Table 2:** Sources of GI bleeding in patients with NOACs or VKA.

	NOACs (*n* = 144)	VKA (*n* = 237)
**Upper GI findings (%)**	51 (35.4)	98 (41.4)
**Esophagus**	8 (5.6)	13 (5.5)
Esophagitis	2 (1.4)	1 (0.4)
Esophageal ulcer	1 (0.7)	1 (0.4)
Mallory-Weiss syndrome	3 (2.1)	7 (3.0)
Esophageal angiodysplasia	0 (0)	1 (0.4)
Esophageal varix	2 (1.4)	3 (1.3)
**Stomach**	38 (26.4)	69 (29.1)
Gastric varix	3 (2.1)	1 (0.4)
Gastric antral vascular ectasia	1 (0.7)	2 (0.8)
Gastric erosion	2 (1.4)	3 (1.3)
Benign gastric ulcer	25 (17.4)	47 (19.8)
Gastric cancer	2 (1.4)	1 (0.4)
Gastric angiodysplasia	2 (1.4)	9 (3.8)
Gastric dieulafoy	1 (0.7)	6 (2.5)
Gastric polypectomy Or endoscopic submucosal dissection bleeding	2 (1.4)	0 (0)
**Duodenum**	5 (3.5)	16 (6.8)
Duodenal ulcer	5 (3.5)	14 (5.9)
Duodenal angiodysplasia	0 (0)	1 (0.4)
Duodenal dieulafoy lesion	0 (0)	1 (0.4)
Duodenitis	0 (0)	0 (0)
**Small bowel findings (%)**	6 (4.2)	16 (6.8)
Inflammatory lesion	2 (1.4)	9 (3.8)
Neoplastic lesion	0 (0)	0 (0)
Vascular lesion	4 (2.8)	6 (2.5)
Others	0 (0)	1 (0.4)
**Lower GI findings (%)**	33 (22.9)	43 (18.1)
**Vascular lesion**	5 (3.5)	13 (5.5)
Hemorrhoid	4 (2.8)	10 (4.2)
Ischemic colitis	1 (0.7)	3 (1.3)
**Anatomic lesion**	8 (5.6)	7 (3.0)
Diverticuli without bleeding	7 (4.9)	4 (1.7)
Diverticuli with current bleeding	1 (0.7)	3 (1.3)
**Inflammatory lesion**	14 (9.7)	10 (4.2)
Rectal ulcer only	8 (5.6)	4 (1.7)
Rectal ulcer with exposed vessel	1 (0.7)	1 (0.4)
Colon ulcer	3 (2.1)	1 (0.4)
Infectious colitis	1 (0.7)	1 (0.4)
Pseudomembranous colitis	1 (0.7)	2 (0.8)
Inflammatory bowel disease	0 (0)	1 (0.4)
**Neoplastic lesion**	6 (4.2)	13 (5.5)
Colon polyp	5 (3.5)	10 (4.2)
Colon cancer	1 (0.7)	3 (1.3)
**U** **nidentified lesion (%)**	54 (37.5)	80 (33.8)

GI, gastrointestinal; NOACs, new oral anticoagulants; VKA, vitamin K agonist.

**Table 3:** Lesion characteristics and location of GI bleeding in patients with NOACs or VKA.

	NOACs (*N* = 90)	VKA (*N* = 157)	*p* Value
**Lesion characteristics (%)**			
Vascular lesion	14 (15.6)	40 (25.5)	0.038
Inflammatory lesion	49 (54.4)	81 (51.6)	0.775
Neoplastic lesion	7 (7.8)	14 (8.9)	0.604
Anatomic lesion & Others *	20 (22.2)	22 (14.0)	0.638
**Location (%)**			
Esophagus	8 (8.9)	13 (8.3)	0.912
Stomach	38 (42.2)	69 (43.9)	0.334
Duodenum	5 (5.6)	16 (10.2)	0.284
Small bowel	6 (6.7)	16 (10.2)	0.090
Colon	33 (36.7)	43 (27.4)	0.460

NOACs, new oral anticoagulants; VKA, vitamin K agonist. * Others category was included diverticular bleeding, Mallory-Weiss syndrome, post polypectomy bleeding, and post endoscopic submucosal dissection bleeding.

**Table 4:** Comparison of clinical outcomes in the patients with NOACs vs. VKA.

Clinical Outcomes	NOACs (%) (*n* = 59)	VKA (%) (*n* = 123)	Multivariate Logistic Regression Analysis *	
			Adjusted OR (95% CI)	*p* Value
Hemodynamic instability at admission	26 (17.7%)	50 (21.6%)	0.81 (0.47–1.37)	0.167
Rebleeding	15 (10.6%)	46 (20.9%)	0.42 (0.22–0.79)	0.007
Need for angiography	11 (8.1%)	12 (5.6%)	1.47 (0.62–3.45)	0.112
Mortality during Hospital day	6 (4.1%)	11 (4.7%)	0.84 (0.30–2.35)	0.729
Need for surgery ^†^	1 (0.7%)	4 (1.7%)	0.82 (0.69–0.98)	0.045

NOACs, new oral anticoagulants; VKA, vitamin K agonist; OR, odds ratio; CI, confidence interval; * These odds ratios and 95% CIs were adjusted for sex, HAS-BLED score; ^†^ The type of surgery in patient with NOACs was distal gastrectomy. The patients with VKA underwent distal gastrectomy (2 cases), small bowel segmental resection (1 case), and right hemicolectomy (1 case).

**Table 5:** The clinical outcomes in the patients associated with different NOACs.

	NOACs	Dabigatran (*n* = 32, 22.2%)	Rivaroxaban (*n* = 72, 50.0%)	Apixaban (*n* = 28, 19.5%)	Edoxaban (*n* = 12, 8.3%)
Outcomes					
Hemodynamic instability at admission		5 (19.3%)	13 (50.0%)	7 (26.9%)	1 (3.8%)
Need for angiography		3 (27.3%)	7 (63.7%)	1 (9.0%)	0 (0.0%)
Need for surgery		0 (0.0%)	1 (100.0%)	0 (0.0%)	0 (0.0%)
Mortality during Hospital day		1 (16.7%)	4 (66.6%)	1 (16.7%)	0 (0.0%)
Rebleeding		2 (13.3%)	9 (60.0%)	4 (26.7%)	0 (0.0%)

NOACs, new oral anticoagulants.

## Data Availability

The datasets generated and/or analyzed during the current study are not publicly available due to our IRB policy.
